# Postictal self‐removal of intracerebral electrodes during stereoelectroencephalography monitoring: A case series

**DOI:** 10.1002/epd2.70175

**Published:** 2026-01-10

**Authors:** Ionuț‐Flavius Bratu, Romain Carron, Julia Makhalova, Stanislas Lagarde, Fabrice Bartolomei

**Affiliations:** ^1^ Assistance Publique—Hôpitaux de Marseille, Epileptology and Cerebral Rhythmology Department, Timone Hospital Marseille France; ^2^ Systems Neuroscience Institute, French National Institute of Health and Medical Research (UMR 1106), Aix‐Marseille University Marseille France; ^3^ Assistance Publique—Hôpitaux de Marseille, Medico‐Surgical Unit of Epileptology, Functional and Stereotactic Neurosurgery, Timone Hospital Marseille France; ^4^ Center for Magnetic Resonance in Biology and Medicine, National Centre for Scientific Research (UMR 7339), Aix‐Marseille University Marseille France

**Keywords:** agitation, confusion, posictal, SEEG, self‐removal

## Abstract

Epilepsy surgery remains the most effective treatment for focal drug‐resistant epilepsy, and stereoelectroencephalography (SEEG) is increasingly used to define the epileptogenic‐zone network (EZN) and guide curative or palliative interventions. While SEEG is considered a safe invasive procedure, adverse events arising during monitoring itself are rarely described. We report three exceptional cases of postictal self‐removal of intracerebral electrodes during SEEG monitoring. Among 591 implanted patients between January 2000 and October 2025 at Timone Hospital, Marseille, three patients (0.5%) met the inclusion criteria. All were young right‐handed men with normal neurocognitive development, focal drug‐resistant epilepsy and no psychiatric comorbidity. Self‐removal occurred during the postictal phase of spontaneous seizures—two following focal‐to‐bilateral tonic–clonic seizures and one after a focal impaired‐awareness seizure—on the second day of monitoring under complete or partial antiseizure medication withdrawal. Postictal behavior was characterized by agitation, wandering, and, in two cases, resistive aggression when nursing staff attempted to intervene. None of the patients sustained neurological sequelae or significant cerebrovascular complications. EZN involved the temporal lobe in all cases. These observations illustrate that postictal confusion, particularly under medication withdrawal, may occasionally manifest as resistive behavior capable of causing self‐harm by means of device manipulation. Awareness of this rare, but potentially hazardous phenomenon, identification of at‐risk patients, and implementation of tailored preventive measures may help improve the safety of invasive epilepsy monitoring.

**Key points**

- Postictal agitation and confusion can exceptionally lead to self‐removal of intracerebral electrodes.
- Such events occurred during partial or complete antiseizure medication withdrawal.
- Seizures originated from temporal lobe–involving epileptogenic networks in all cases.
- Enhanced supervision and tailored safety measures are essential during invasive EEG monitoring.

## 1 INTRODUCTION

Epilepsy surgery remains the most effective treatment for focal drug‐resistant epilepsy (fDRE).^1^ When noninvasive presurgical investigations yield concordant data, patients may proceed directly to resective surgery; however, if the results are inconclusive or discordant, invasive intracranial electroencephalography is warranted.^2^ In this regard, stereoelectroencephalography (SEEG) has gained increasing adoption worldwide compared with subdural grid explorations, owing to its superior safety profile, higher postsurgical seizure‐freedom rates, and its ability to guide neuromodulation therapies and minimally invasive ablative procedures such as SEEG‐guided radiofrequency thermocoagulation (RFTC).^3^ Throughout the video‐SEEG monitoring, continuous patient surveillance by specialized staff in a dedicated environment is essential.^4^ Antiseizure medications (ASM) are often tapered to increase the likelihood of recording spontaneous seizures.^5^ While this approach is generally safe, ASM reduction undeniably increases the risk of adverse events.^6^

The postictal state—the transient brain dysfunction following seizure termination^7^ —encompasses a broad spectrum of neurobehavioral alterations, including confusion, agitation, disinhibition, and, more rarely, aggressive behaviors.^8^, ^9^, ^10^ Although postictal agitation and aggression are well‐recognized, they rarely result in self‐injury or manipulation of medical devices. Within the context of invasive monitoring, however, such behaviors may lead to mechanical disruption or self‐removal of SEEG electrodes. Despite extensive literature on SEEG safety, most reported complications and adverse events concern implantation‐explantation^11^ or RFTC procedures,^12^ whereas complications and adverse events arising during monitoring remain scarcely documented.^13^

Here, we describe a case series of patients who, during postictal confusion following spontaneous seizures, pulled out their intracerebral electrodes during ongoing SEEG monitoring.

## 2 METHODS

### 2.1 Case series

Patients were selected from the database of the Epileptology Department, Timone Hospital, Marseille, France, according to the following criteria: (1) SEEG exploration, (2) occurrence of an adverse event during monitoring, and (3) self‐removal of implanted electrodes. Among 591 patients who underwent SEEG implantation between January 2000 and October 2025, three met these criteria (0.5%). All patients were male, aged 24–31 years (mean 27.0 ± 3.61) (Table 1).

**TABLE 1:** Patients' electro‐clinical characteristics.

ID	Sex	Age at seizure onset	Age at SEEG	Education level	IQ	Postictal history	IRM	SEEG‐defined EZN	ASM at the time of the event	ASM tapering at the time of the event	Type of seizure followed by self‐electrode removal
P1	M	19	31	Master's	88	Aphasia Psychosis (visual–auditory hallucinations; delusions of influence)	Normal	Left mesial temporal	CBZ 800 mg PER 4 mg BRV 50 mg	CBZ 0 mg PER 0 mg BRV 0 mg	Focal impaired consciousness seizure with observable manifestations
P2	M	9	24	Post‐secondary vocational qualification	95	Aphasia Asthenia	Normal	Left posterior superior temporal gyrus	CBZ 1000 mg BRV 50 mg	CBZ 0 mg BRV 0 mg	Focal‐to‐bilateral tonic–clonic
P3	M	4	26	Bachelor's	74	Prolonged agitation with wandering	Right anterior temporal lobectomy and gamma‐knife scars	Right frontal operculum—posterior superior temporal gyrus	LEV 2000 mg CBZ 800 mg LCS 250 mg TPM 25 mg	LEV 0 mg CBZ 0 mg LCS 250 mg TPM 25 mg	Focal‐to‐bilateral tonic–clonic

Abbreviations: ASM, antiseizure medication; BRV, brivaracetam; CBZ, carbamazepine; EZN, epileptogenic‐zone network; LCS, lacosamide; LEV, levetiracetam; PER, perampanel; SEEG, stereoelectroencephalography; TPM, topiramate.

**FIGURE 1:**
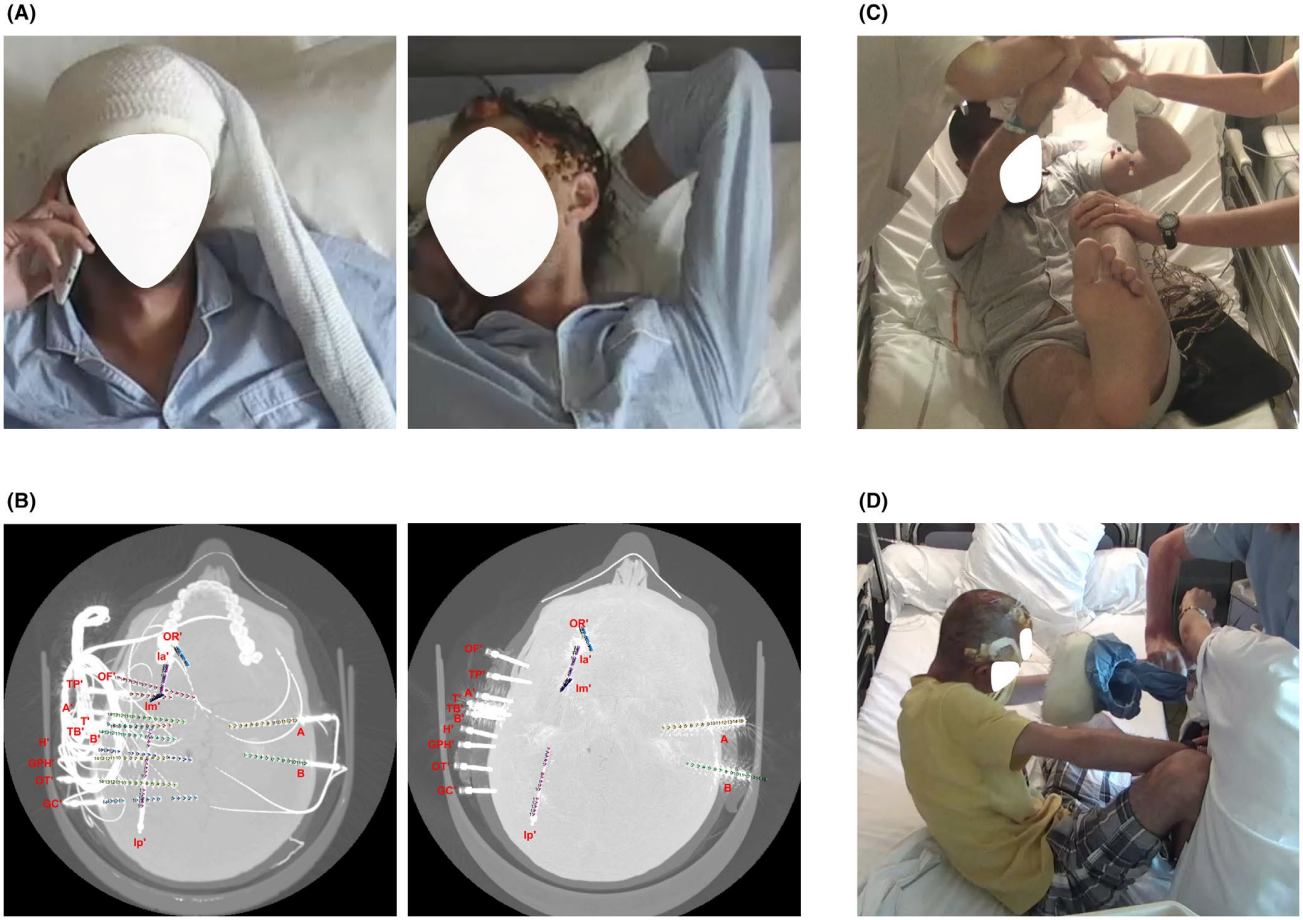
Behavioral and imaging aspects of postictal self‐electrode removal during SEEG monitoring. (A) Patient 1 before (left) and after (right) self‐removal of intracerebral electrodes. (B) Post‐implantation (left) and post self‐removal (right) head computed tomography scans of Patient 1. After self‐extraction, only the anchors of the left electrodes with orthogonal trajectories are visible, while the oblique and right‐sided electrodes appear displaced. (C) Patients 2 and (D) Patient 3 during the postictal period exhibiting agitation and resistive behavior while pulling their electrodes, as nursing staff intervene.

At implantation, Full‐Scale IQs (Wechsler Adult Intelligence Scale) ranged 74–95 (mean 85.7 ± 10.7). All patients had at least a post‐secondary education, no psychiatric history and were not receiving any psychotropic medication. Self‐removal occurred during the postictal phase of focal‐to‐bilateral tonic–clonic seizures in two cases (P2, P3) and after a focal impaired‐awareness seizure evolving into a bilateral propagation network in one (P1) (Figure 1). All incidents occurred on the second day of video‐SEEG monitoring: P1 and P2 under complete ASM withdrawal, P3 under partial tapering. Before these events, P1 had experienced postictal psychosis and P3 postictal agitation with wandering. Mean proportion of self‐removed electrodes was 39% (1016 for P1, 3/9 for P2 and 2/9 for P3). Following self‐removal, all remaining implanted electrodes and anchors were explanted under imaging control (Figure 1A,B). Post‐explantation imaging confirmed the absence of retained electrode fragments or insertion screws and revealed no clinically significant complications. Findings were limited to minor, asymptomatic changes—namely a small increase in preexisting periprocedural pneumocephalus (P1), a small de novo asymptomatic frontal hemorrhage with mild pneumocephalus (P2) and no abnormalities in P3. No patient reported any neurological deficit or subsequent deterioration in quality of life.

### 2.2 Case‐by‐case presentation

**FIGURE 2:**
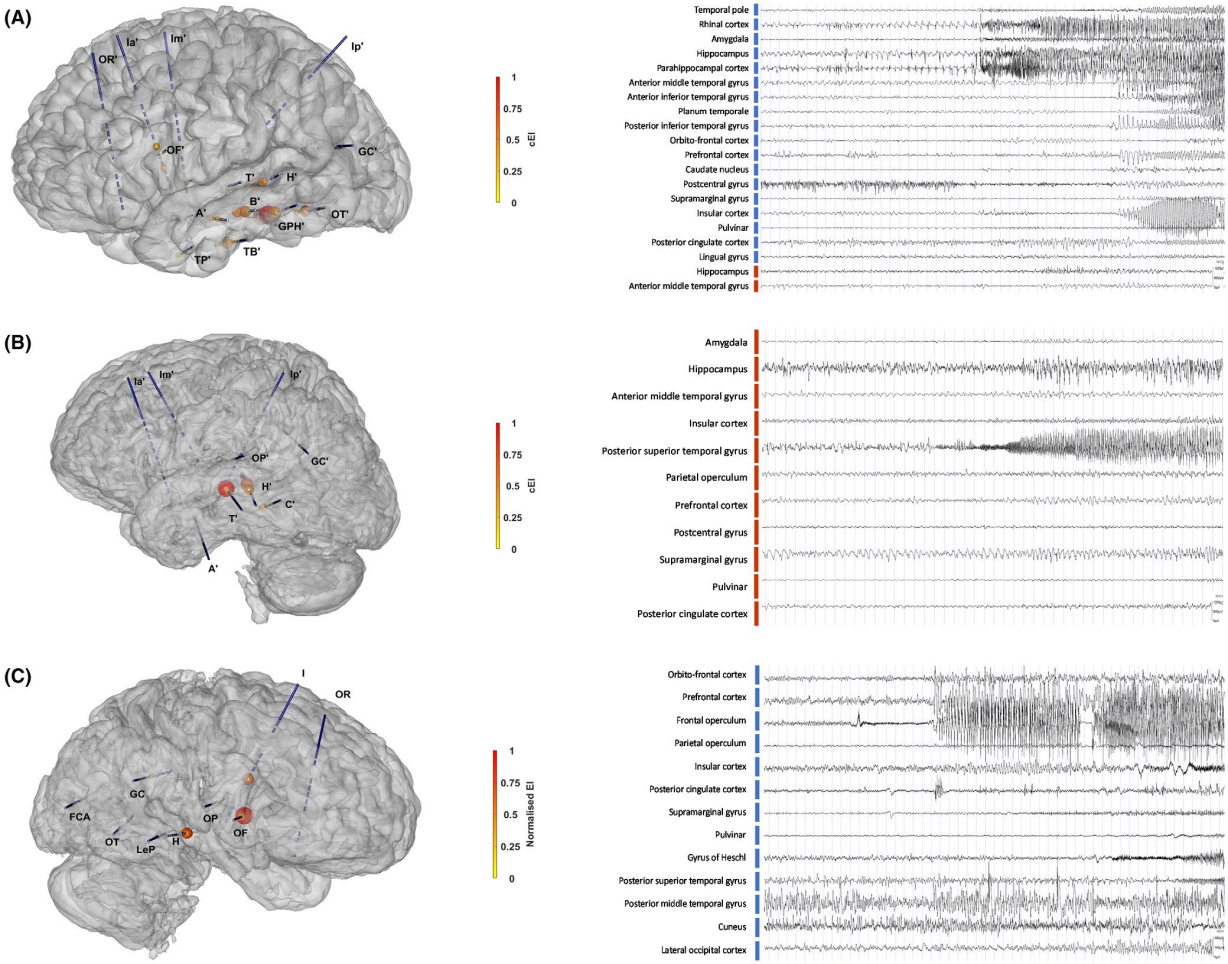
Organization of the epileptogenic‐zone network (EZN) in the seizures followed by self‐electrode removal. (A–C, left images) SEEG exploration reconstructions with normalized c/EI values: Depth electrode positions are reconstructed by combining data from the patient's post‐implantation cerebral CT scan with a 3D inflated cerebral mesh derived from the pre‐implantation cerebral T1‐weighted MRI scan. Normalized c/EI values range from 0 (no epileptogenicity, bright yellow) to 1 (maximum epileptogenicity, dark red), depicted on the mesh as colored blobs positioned on electrode contacts. (A–C, right images) Seizure onset and primary seizure organization as depicted on the stereoelectroencephalography traces; blue bars—left hemisphere electrodes and brown bars—right hemisphere electrodes. Panel A—left mesial temporal EZN in Patient 1, Panel 2—left posterior superior temporal gyrus EZN in Patient 2 and Panel C—right frontal‐temporal EZN in Patient 3.

*Patient 1 (P1)* 31‐year‐old right‐handed man with normal neurocognitive development, focal drug‐resistant epilepsy since the age of 19 years old, without identifiable epilepsy risk factors. Seizures occurred monthly, often in clusters and frequently had bilateral tonic–clonic evolution. Semiology involved an ecstatic aura, impaired awareness, gestural automatisms, and mixed aphasia extending into the postictal period—lasting up to 30 min—and comprising jargon aphasia with transient recovery of a foreign language before regaining fluency in his native language. He experienced one episode of postictal psychosis with hallucinations and delusions of influence after a focal‐to‐bilateral tonic–clonic seizure, resolving within weeks under risperidone. Video‐EEG recordings showed seizure onset in the left fronto‐temporal derivations. Cerebral MRI showed no macroscopic abnormalities, while cerebral PET‐CT demonstrated left insular–temporal hypometabolism. WAIS indicated stronger visual–perceptual reasoning relative to verbal comprehension. SEEG implantation mainly targeted a left temporal–insular hypothesis. During full ASM withdrawal (Table 1), one spontaneous seizure was recorded, followed by postictal self‐removal of left orthogonal electrodes. At seizure onset, while speaking on the phone, he was able to announce to the interlocutor that he was having a seizure, then developed left‐hand dystonia, gestural automatisms, left head deviation, loss of awareness, and left lower‐limb automatisms, followed by clonic movements of the right hemiface, right head version, and bilateral upper‐limb, laryngeal, and abdominal clonia. After seizure termination, he became agitated, left the bed, and—despite attempts by the nursing staff to intervene—pulled out 10 out of the 16 implanted electrodes (Figure 1A,B). He subsequently returned to bed, appeared calm and unaware, and exhibited oro‐alimentary automatisms. Correct interaction with the nurses was possible at 13 min postictally. The remaining implanted electrodes (six out of sixteen) were removed the following day, and post‐explantation imaging revealed no complications apart from a minor increase in periprocedural pneumocephalus. Visual SEEG analysis, complemented by quantifications using the Connectivity Epileptogenicity Index,^14^ localized the EZN to the left anterior mesial temporal region (Figure 2A).

*Patient 2 (P2)*, a 24‐year‐old right‐handed man with normal neurocognitive development, has focal drug‐resistant epilepsy since the age of 9 years old, without identifiable epilepsy risk factors. Seizures were mainly triggered by environmental noise, to which he showed marked hypersensitivity—particularly high‐pitched or repetitive sounds such as running water. Seizure semiology consisted of an unpleasant internal sensation that progressively intensified, followed by autonomic and motor manifestations including tachycardia, facial flushing, and hypermotor behavior. At onset, he typically remained aware, sat down, and attempted to stop the seizure if it was triggered by noise by covering his ears and screaming. Postictally, he presented brief word‐finding difficulty and fatigue. Video‐EEG recordings were non‐localizing but demonstrated predominant propagation in the left hemisphere. Cerebral MRI showed no macroscopic abnormalities, while cerebral PET‐CT showed left anterior temporal and frontal‐opercular hypometabolism. WAIS indicated comparable verbal‐comprehension and perceptual‐reasoning scores. A first SEEG exploration identified an EZN involving the left posterior superior temporal gyrus within the language‐dominant hemisphere. SEEG‐guided RFTC at this level resulted in transient anomia but a marked reduction in seizure frequency. Given the partial benefit of RFTC and the proximity of the EZN to eloquent cortex, a second SEEG exploration was undertaken to guide additional thermocoagulation. During this second SEEG implantation, under complete ASM withdrawal (Table 1), one spontaneous seizure was recorded, followed by postictal self‐removal of electrodes. The seizure arose from sleep, beginning with leftward turning, grimacing, and left‐hand dystonia, followed by attempts to cover his ears and hyperventilate in an effort to abort the episode. Axial anteroposterior rocking movements ensued, progressing to bipedal automatisms, right‐sided head version, and bilateral tonic–clonic generalization. After seizure termination, he remained asthenic with labored breathing and oro‐alimentary automatisms, minimally responsive to testing. Nine minutes postictally, while the nursing staff attempted to administer medication, he became increasingly agitated—pushing them away and pulling at the electrode wires (Figure 1C). The behavior escalated; he left the bed, continuing to pull the electrodes and by 15 min postictally had removed three out of nine implanted electrodes and displaced or sectioned the wires of six others. He then gradually calmed down and fell asleep, awakening without neurological deficit. The remaining electrodes (six out of nine) were removed the following day, and post‐explantation imaging revealed only an infracentimetric frontal asymptomatic hemorrhage and minor pneumocephalus. Visual SEEG analysis, complemented by quantifications using the Connectivity Epileptogenicity Index,^14^ confirmed seizure onset within the previously identified EZN (Figure 2B).

*Patient 3 (P3)* 26‐year‐old right‐handed man with normal neurocognitive development and focal drug‐resistant epilepsy since the age of 4 years old, with identifiable risk factors including congenital toxoplasmosis. Cerebral MRI showed no macroscopic abnormalities, while cerebral PET‐CT demonstrated right mesial‐polar and temporo‐perisylvian hypometabolism. Video‐EEG recordings were concordant with right frontal‐temporal semiology and ictal onset. Interictal SPECT suggested right mesial temporal involvement and WAIS indicated weaker visual–perceptual reasoning relative to verbal comprehension. An initial SEEG exploration delineated an EZN encompassing the right mesial and lateral temporal regions. The patient subsequently underwent an anterior temporal lobectomy, including the neocortical superior temporal gyrus and Heschl's gyrus, which yielded no significant improvement, followed by gamma‐knife treatment of the posterior temporal neocortex and residual Heschl's gyrus, resulting in marked seizure reduction. At the time of the second SEEG, seizure semiology included bilateral elementary auditory hallucinations followed by an epigastric sensation of fear and *déjà vu*, *déjà vécu* or *déjà rêvé* experiences, progressing to impaired awareness, bilateral eyelid clonia and psychomotor slowing. Seizures were often triggered by non‐specific sounds or music. The postictal phase was characterized by intense and prolonged agitation with wandering behavior, often triggered by non‐specific sounds or music. Video‐EEG recordings confirmed a right temporal seizure onset, prompting a second SEEG exploration. During this video‐SEEG monitoring, while being on partially tapered ASM quadritherapy, several auras were recorded, as well as one focal seizure manifesting with bilateral gestural and oro‐alimentary automatisms, left‐hand dystonia and secondary bilateral tonic–clonic generalization. After seizure termination, the patient appeared asthenic, with labored breathing and minimal responsiveness to postictal testing. Approximately 1 min later, he became increasingly agitated—pushing staff away and pulling at the electrode wires. The behavior escalated to resistive aggression; he left the bed, continued pulling the electrodes and began wandering through the ward despite attempts by two nurses to restrain him (Figure 1D). About 3 min postictally, he followed the staff back to his room, but he did not interact normally for about half an hour. As a result of the postictal behavior, two of the nine implanted electrodes were removed and five had their wires sectioned. The remaining electrodes were explanted the following day and post‐explantation imaging revealed no complication. Visual SEEG analysis, complemented by quantifications using the Epileptogenicity Index,^14^ confirmed seizure onset within a right frontal‐temporal EZN (Figure 2C).

## 3 DISCUSSION

To the best of our knowledge, this case series represents the first report of postictal self‐removal of intracerebral electrodes during SEEG monitoring, an exceptional adverse event of invasive monitoring. This phenomenon illustrates how postictal behavioral dysregulation, particularly in the setting of SEEG exploration and under ASM withdrawal, may lead to electrode manipulation or self‐removal, underscoring the need for preventive safety measures. Although such adverse events occurred in only 0.5% of our historical SEEG cohort—and proportionally less when considering patients who underwent multiple SEEG explorations—their potential to cause serious harm to patients and significant physical or psychological distress to monitoring personnel warrants explicit reporting. Fortunately, none of our patients experienced any complications related to self‐removal of the electrodes (no symptomatic hemorrhage, infection, clinically significant pneumocephalus, or neurological deficit). This is probably due to the fact that when the electrodes are pulled, their trajectory remains constrained by the anchors, thereby limiting the risk of parenchymal injury. However, the possibility of electrode fracture cannot be theoretically excluded. In addition, traction on the electrode can compromise the integrity of the anchoring system, creating an abnormal communication between the intracranial compartment and the external environment, thereby predisposing to CSF leakage and requiring prompt recognition and management. Finally, premature electrode self‐removal compromises the quality of the SEEG investigation, resulting in incomplete quantification of epileptogenic biomarkers and limited functional mapping, while also preventing the possibility of performing SEEG‐guided thermocoagulation.

Several factors may have contributed to these events, including a history of postictal agitation or psychosis, psychiatric comorbidities, ASM tapering, the type of EZN, seizure characteristics and the level of monitoring. Only P1 had a previous episode of postictal psychosis, while none of the patients had a chronic psychiatric disorder or were taking psychotropic medication. P3 had a known history of prolonged postictal confusion with agitation and wandering, whereas P2 typically exhibited postictal asthenia. Importantly, all three self‐removal events occurred during ASM tapering, on the second day of the monitoring period; however, only P1 and P2 were under complete withdrawal. Evers et al.^15^ had previously shown that a psychiatric history, whether epilepsy‐related or not, is the main predictor of behavioral and psychiatric dysregulation during invasive monitoring, while the ASM tapering rate was not significantly associated. ASM tapering might have increased the spatial extent of seizure organization and the severity of postictal electro‐clinical disturbances. Regarding seizure localization, all patients had EZNs involving the temporal regions, which aligns with literature indicating that postictal psychiatric and behavioral complications are more common in temporal lobe epilepsy.^16^

In terms of phenomenology, all three patients exhibited postictal agitation, wandering, with electrode manipulation appearing as part of a broader behavioral drive to move or escape. Patients 2 and 3, however, displayed clear resistive–aggressive behavior, actively pushing away nursing staff attempting to intervene. Kanemoto et al.^9^ reported that resistive violence occurred only during postictal confusion (3.0% of episodes) and is thought to represent an unconscious defensive response to restraint, whereas well‐directed violence was observed in 22.8% of postictal psychosis episodes, but only 0.7% of postictal confusion episodes. All our patients were under continuous supervision at the time of the incident, with at least two members of the nursing staff present and immediate postictal care initiated. In P1, cyamemazine was pre‐prepared in anticipation of agitation due to his history of postictal psychosis and complete ASM withdrawal. Considering the combination of vigorous resistive behavior and staff intervention, it is plausible that avoiding direct physical contact during the initial phase of recovery might reduce such behavioral risks in selected cases. Nevertheless, given that two of the three seizures evolved to bilateral tonic–clonic activity, airway protection and patient safety must take priority over the rare possibility of self‐removal.

Altogether, our series highlights that postictal behavioral dysregulation during SEEG monitoring may exceptionally manifest as “flight” and resistive behavior capable of causing self‐harm by means of device manipulation, even in the absence of psychiatric comorbidity or psychosis. Early recognition of such behavioral patterns, preventive measures for high‐risk patients (e.g., those with a history of postictal agitation) and systematic documentation of such events may improve the safety of invasive epilepsy monitoring. One question that may arise concerns the type of dressing that could more effectively prevent self‐removal of electrodes. Although some authors have advocated a cast (US) or plaster‐cast (UK) dressing around the electrode bundles, we do not recommend this approach. For such a dressing to be effective, it must be applied tightly, which may substantially increase postoperative headaches and still does not reliably prevent electrode extraction. Moreover, the rigid and sharp edges of a cast can sever lead wires, and at some point these wires must remain accessible, meaning that traction—and therefore risk—cannot be fully eliminated.

Instead, nurses' vigilance and rapid intervention remain the most effective preventive measures. Nevertheless, in cases of marked postictal agitation, electrode manipulation or self‐removal may remain unavoidable despite optimal supervision.

## AUTHOR CONTRIBUTIONS

I‐F.B.: Conceptualization, Data curation, Formal analysis, Investigation, Methodology, Project administration, Software, Validation, Visualization, Writing—original draft, Writing—review and editing; R.C.: Data curation, Writing—review and editing; J.M.: Data curation, Writing—review and editing; S.L.: Data curation, Writing—review and editing; F.B.: Conceptualization, Data curation, Funding acquisition, Investigation, Methodology, Project administration, Resources, Supervision, Validation, Writing—original draft, Writing—review and editing.

## CONFLICT OF INTEREST STATEMENT

The authors declare no conflict of interest to disclose.

## PATIENT CONSENT

All patients have given informed written consent and this study was approved by Assistance Publique—Hôpitaux de Marseille.

**Test Yourself**

1. Which of the following postictal behaviors, observed in SEEG monitoring, may reflect a defensive or “flight” reaction during confusion?

- Stereotyped automatisms.
- Resistive aggression and escape attempts.
- Verbal hallucinations.
- Perseverative speech.

2 Postictal behavioral dysregulation, including agitation and resistive behavior, is most frequently associated with the epileptogenic‐zone network involving which lobe?

- Frontal lobe.
- Parietal lobe.
- Temporal lobe.
- Occipital lobe.

3 In all patients who self‐removed their SEEG electrodes, which factor was common at the time of the incident?

- Presence of a focal cortical dysplasia.
- Rapid or complete antiseizure medication withdrawal.
- Long‐standing psychiatric comorbidity.
- Sleep deprivation during monitoring.

*Answers may be found in the* *supporting information*

## Supporting information


Data S1.

## Data Availability

The data that support the findings of this study are available from the corresponding author upon reasonable request.

## References

[1] Engel J . What can we do for people with drug‐resistant epilepsy? Neurology. 2016;87(23):2483–2489. 27920283 10.1212/WNL.0000000000003407 PMC5177675

[2] Frauscher B , Mansilla D , Abdallah C , Astner‐Rohracher A , Beniczky S , Brazdil M , et al. Learn how to interpret and use intracranial EEG findings. Epileptic Disord. 2024;26(1):1–59. 38116690 10.1002/epd2.20190

[3] Ryvlin P . SEEG in 2025: progress and pending challenges in stereotaxy methods, biomarkers and radiofrequency thermocoagulation. Curr Opin Neurol. 2025;38(2):111–120. 39927419 10.1097/WCO.0000000000001351 PMC11888833

[4] Isnard J , Taussig D , Bartolomei F , Bourdillon P , Catenoix H , Chassoux F , et al. French guidelines on stereoelectroencephalography (SEEG). Neurophysiol Clin. 2018;48:5–13. 29277357 10.1016/j.neucli.2017.11.005

[5] Chassoux F , Navarro V , Catenoix H , Valton L , Vignal JP . Planning and management of SEEG. Neurophysiol Clin. 2018;48(1):25–37. 29254835 10.1016/j.neucli.2017.11.007

[6] van Griethuysen R , van Asch CJJ , Otte WM , Lamberink H , Sander JW , Bourez‐Swart MD , et al. Antiseizure medication reduction in long‐term video‐electroencephalographic monitoring for presurgical evaluation: a multicenter safety and efficacy analysis. Epilepsia. 2025;1:1–11. 10.1111/epi.18572 40709605

[7] Fisher RS , Engel JJ . Definition of the postictal state: when does it start and end? Epilepsy Behav. 2010;19(2):100–104. 20692877 10.1016/j.yebeh.2010.06.038

[8] Kanner AM , Soto A , Gross‐Kanner H . Prevalence and clinical characteristics of postictal psychiatric symptoms in partial epilepsy. Neurology. 2004;62(5):708–713. 15007118 10.1212/01.wnl.0000113763.11862.26

[9] Kanemoto K , Tadokoro Y , Oshima T . Violence and postictal psychosis: a comparison of postictal psychosis, interictal psychosis, and postictal confusion. Epilepsy Behav. 2010;19(2):162–166. 20727827 10.1016/j.yebeh.2010.06.018

[10] Bartolomei F , Lagarde S , Lambert I , Trébuchon A , Villalon SM , McGonigal A , et al. Brain connectivity changes during ictal aggression (a strangulation attempt). Epileptic Disord. 2017;19(3):367–373. 28830845 10.1684/epd.2017.0925

[11] Mullin JP , Shriver M , Alomar S , Najm I , Bulacio J , Chauvel P , et al. Is SEEG safe? A systematic review and meta‐analysis of stereo‐electroencephalography–related complications. Epilepsia. 2016;57(3):386–401. 10.1111/epi.13298 26899389

[12] Bregianni M , Pizzo F , Lagarde S , Makhalova J , Trebuchon A , Carron R , et al. Psychiatric complications following SEEG‐guided radiofrequency thermocoagulations in patients with drug‐resistant epilepsy. Epilepsy Behav. 2024;156:1872. 10.1016/j.yebeh.2024.109806 38677102

[13] Soncin LD , Arthuis M , Scholly J , Carron R , McGonigal A , Pizzo F , et al. “Plok‐plok” syndrome: posttraumatic stress disorder following an SEEG thermocoagulation and direct electrical stimulation procedure. Epileptic Disord. 2023;25(3):390–396. 10.1002/epd2.20023 36939714

[14] Balatskaya A , Roehri N , Lagarde S , Pizzo F , Medina S , Wendling F , et al. The “connectivity epileptogenicity index” (cEI), a method for mapping the different seizure onset patterns in StereoElectroEncephalography recorded seizures. Clin Neurophysiol. 2020;131(8):1947–1955. 32622336 10.1016/j.clinph.2020.05.029

[15] Evers MEA , Nelissen J , Vlooswijk MCG , van Kranen‐Mastenbroek VHBM , Leentjens AFG , Rouhl RPW , et al. Risk factors for behavioral and psychotic dysregulation at the epilepsy monitoring unit in patients with intracranial electrodes. Epilepsy Behav. 2023;148:109448. 37776593 10.1016/j.yebeh.2023.109448

[16] Clancy MJ , Clarke MC , Connor DJ , Cannon M , Cotter DR . The prevalence of psychosis in epilepsy; a systematic review and meta‐analysis. BMC Psychiatry. 2014;14(1):1–9. 10.1186/1471-244X-14-75 PMC3995617 24625201

