# Supplementary material for: Postictal self‐removal of intracerebral electrodes during stereoelectroencephalography monitoring: A case series
Source: Epileptic Disord. 2026 Jan 10;28(2):488–95. doi: 10.1002/epd2.70175 (PMC13084203; doi:10.1002/epd2.70175)
Supplement: Supplementary file 1 — Data S1. [file EPD2-28-488-s001.docx]

1. **Correct answer:** B

2. **Correct answer:** C

**3. Correct answer:** B
